# Stigma and posttraumatic growth among COVID-19 survivors during the first wave of the COVID-19 pandemic in Malaysia: a multicenter cross-sectional study

**DOI:** 10.3389/fpsyt.2023.1152105

**Published:** 2023-04-24

**Authors:** Nazirah Azman, Nik Ruzyanei Nik Jaafar, Mohammad Farris Iman Leong Bin Abdullah, Nur Iwana Abdul Taib, Nurul Ain Mohamad Kamal, Muhammad Najib Abdullah, Siti Nordiana Dollah, Mohd Shahrir Mohamed Said

**Affiliations:** ^1^Department of Psychiatry, Faculty of Medicine, Universiti Kebangsaan Malaysia, Kuala Lumpur, Malaysia; ^2^Hospital Canselor Tuanku Muhriz (HCTM), Jalan Yaacob Latif, Bandar Tun Razak, Kuala Lumpur, Malaysia; ^3^Department of Community Health, Advanced Medical and Dental Institute, Universiti Sains Malaysia, Kepala Batas, Pulau Pinang, Malaysia; ^4^Department of Psychological Medicine, Faculty of Medicine and Health Sciences, Universiti Malaysia Sarawak, Kota Samarahan, Malaysia; ^5^Department of Psychiatry and Mental Health Hospital, Sungai Buloh, Selangor, Malaysia; ^6^Department of Psychiatry Hospital Angkatan Tentera Tuanku Mizan, Kuala Lumpur, Malaysia; ^7^Department of Medicine, Faculty of Medicine, Universiti Kebangsaan Malaysia, Kuala Lumpur, Malaysia

**Keywords:** posttraumatic growth, stigma, COVID-19, psychological trauma, social stigma

## Abstract

**Background:**

Contracting COVID-19 can cause negative and distressing psychological sequelae, but traumatic stressors may also facilitate the development of positive psychological change beyond an individual’s previous level of adaptation, known as posttraumatic growth (PTG). As a result, studies have investigated the negative effects of COVID-19 on mental health, but data on PTG among patients who have recovered from COVID-19 remains limited. This study aims to evaluate the level of PTG and its associations with stigma, psychological complications, and sociodemographic factors among COVID-19 patients 6 months post-hospitalization.

**Method:**

A cross-sectional online survey of 152 COVID-19 patients was conducted after 6 months of being discharged from Hospital Canselor Tuanku Muhriz, MAEPS Quarantine Center, or Hospital Sungai Buloh, Malaysia. Patients completed a set of questionnaires on sociodemographic and clinical data. The Posttraumatic Growth Inventory (PTGI-SF) was used to assess the level of PTG, the Kessler Psychological Distress (K6) was used to measure the degree of psychological distress, the General Anxiety Disorder-7 (GAD-7) was used to evaluate the severity of anxiety symptoms, the Patient Health Questionnaire (PHQ-9) was used to assess the severity of depression symptoms, and the Explanatory Model Interview Catalog Stigma Scale (EMIC-SS) was used to record the degree of perceived stigma toward COVID-19.

**Results:**

The median PTGI SF score of the respondents was 40.0 (Interquartile range 16.0). Multivariable general linear model with bootstrapping (2,000 replications) revealed factors that significantly predicted PTG, which were at the higher level of the perceived stigma score, at 37 (*B* = 0.367, 95% CI = 0.041 to 0.691, *p* = 0.026), among the Malay ethnicity (*B* = 12.767, 95% CI 38 = 7.541 to 17.993, *p* < 0.001), retirees (*B* = −12.060, 95% CI = −21.310 to −2.811, *p* = 0.011), and those with a history of medical illness (*B* = 4.971, 95% CI = 0.096 to 9.845, *p* = 0.046).

**Conclusion:**

Experiencing stigma contributed to patients’ PTG in addition to psychosocial factors such as ethnicity, history of medical illness, and retirement.

## 1. Introduction

The world experienced its first pandemic of modern times over a decade ago, with the 2009 H1N1 swine flu outbreak. This was followed by the COVID-19 pandemic in 2020, caused by the SARS-CoV-2 virus, which mutated rapidly into different variants over time. Moreover, asymptomatic individuals were unaware of the possibility of them transmitting the disease. It slowly became obvious that COVID-19-related mortalities were growing in number, especially during the initial phase and when vaccinations were yet to be developed or made available (1). In addition, the course of the illness became unpredictable as some continued to experience what was later referred to as long COVID-19, where symptoms lingered long after a patient no longer produced positive outcomes on test kits. Long COVID-19 was found to occur in the population regardless of age or severity of the initial symptoms (2).

The unique features of COVID-19 posed significant psychosocial impacts on the community as compared to other diseases. Many became more concerned about the safety measures during the pandemic, especially handwashing and social distancing. In addition, self-quarantine and lockdowns were adopted in many countries across the world, including Malaysia. There was also a surge in the hoarding of daily necessities such as food, drinking water, and toilet paper during the pandemic (3, 4). In other words, mankind was pressured to survive during the pandemic, and survival became a matter of major concern in every household.

With the constant pressure to survive, COVID-19 was a traumatic stressor capable of bringing about Post Traumatic Stress Disorder (PTSD) symptomology (5). An increase in PTSD-like symptoms was reported among participants who had COVID-19 infections or when their family members became infected. Moreover, those with no direct contact with the virus were also anxious about becoming infected at any point. Many members of the public lost their source of income, faced lockdown directives, and experienced changes in their financial dependency during the pandemic (5).

Multiple studies have concluded that having experienced COVID-19 poses a high level of negative psychological sequelae; leading to depression, anxiety, and insomnia (6, 7). Fear of the unknown and having limited knowledge regarding COVID-19 additionally culminated in anxiety and distress among the public (8). A local study among healthcare workers found that frontline workers were inclined to be highly cautious toward COVID-19, and thus, were significantly predicted to have higher anxiety scores (9). Concurrently, misleading information caused confusion and increased public anxiety (10).

In addition, the extreme waves of fear of contracting COVID-19 led to prejudicial behavior and discrimination within several communities, particularly among healthcare workers involved in departments managing COVID-19 patients (11). There was also a high level of perceived stigma toward people infected with COVID-19 and their contacts (12). Stigmatization of specific ethnic groups or those living in certain high-risk locations led to delays in or dismissal of seeking medical help (13).

On the contrary, this crisis led to the development of Posttraumatic Growth (PTG). According to Tedeschi and Calhoun (14), PTG arises through a positive adaptation process, in which people cognitively reappraise their traumatic experience to generate a positive psychological change to a level beyond the pre-traumatic state (15). The five main domains of PTG are having an enhanced perception of personal strength, openness to spiritual issues, finding new possibilities in life, greater appreciation of life, and being able to relate to others.

PTG is based on the affective-cognitive processing model, whereby when a traumatic event is encountered, a person’s assumptive world of self, others, and the surroundings initially shatters. This leads to emotional distress, but the individual learns to understand the meaning of the traumatic experience and successfully undergoes transformational growth, supported by the assimilation and accommodation process (16). Longitudinal studies have demonstrated that PTG does not occur immediately post-trauma but develops 6 months later (17). As such, individuals surviving a traumatic experience would struggle, sometimes for years, before being able to find meaning and move on with life (18, 19).

According to existing studies, in the presence of positive reappraisal coping as the intermediate variable, a perceived stigma can partially and indirectly affect the development of PTG (20). In another longitudinal study among HIV-positive patients, PTG was found to be predicted by an internalized stigma and other factors including rumination, perceived past resilience, positive thinking, and emotional expression (21). PTG has also been shown to have a positive effect among HIV patients moderated to receive support (22). Adopting a more positive attitude also promotes better adherence to treatment (23), hence the reduction in the rate of HIV progression (24).

Despite the high mortality rate of COVID-19 infections and the risk of those infected becoming traumatized, data on the level of PTG among COVID-19 patients are scarce. To date, the only available research exploring PTG among COVID-19 patients was conducted in China (25, 26). Furthermore, although previous studies have demonstrated that COVID-19 patients experienced stigma and psychological distress (6, 7, 12), the association between these components and PTG among COVID-19 patients is yet to be explored.

Therefore, this study aims to evaluate the level of PTG and its association with stigma, psychological complications, and sociodemographic factors among COVID-19 patients 6 months post-hospitalization. It is hypothesized that COVID-19 survivors who perceived greater stigma would be more likely to develop PTG. The study was conducted during the phase when vaccinations were not available, national lockdowns were enforced, and COVID-19 was spreading rapidly. However, at the time of writing, many countries have survived the peak of the pandemic and are transitioning toward the endemic phase. At this point, many are also more receptive to COVID-19, but we find importance in reflecting on the COVID-19 experience, especially for patients with a profile that means they are likely to develop PTG. Subsequently, the data could provide a basis for appropriate intervention in the future to enhance PTG among patients following pandemic-related trauma.

## 2. Methods

### 2.1. Study design, setting, and population

A cross-sectional study was conducted among patients with confirmed COVID-19 between 1st June 2020 and 31st August 2020. Participants were recruited after they were discharged from any of the three COVID-19 treatment centers, i.e., Hospital Canselor Tuanku Muhriz (HCTM), Hospital Sungai Buloh, and Malaysia Agro Exposition Park Serdang (MAEPS) Quarantine Center via consecutive sampling. These hospitals were among the initially designated centers for handling COVID-19 cases in Malaysia (27). The MAEPS center was selected as it was the first Low-Risk COVID-19 Integrated Quarantine and Treatment Center (PKRC) in Malaysia (28).

All accessible subjects were approached to be recruited in the study and further informed regarding the study via email, phone call, and text message. The eligibility criteria included (i) being aged 18 years old and above, (ii) having been hospitalized due to COVID-19 infection (confirmed via PCR) approximately 6 months prior, and (iii) being able to read and write in English or the Malay language. Foreigners and those who were medically and/ or mentally unstable during their hospitalization were excluded from the study. Participants were engaged by on-site researchers (doctors) during their hospitalization, who assessed the suitability and stability of the participant’s general and mental health condition for inclusion in the study. All participants provided their informed consent after reading the survey information sheet in an online Google Form and were assured anonymity and data confidentiality. They were then directed to complete the self-report questionnaire, which takes around 20 min to complete.

The sample size needed for the study (130 subjects) was calculated based on the following formula: *n* = Z1 − α/2 × σ2/∆2, where n represents the total estimated sample size; Z1 − α/2 is the desired confidence interval value, which was selected at 95%, with a critical value of 1.96; σ is the standard deviation (SD), which was 6.5 based on a study of the prevalence of PTG in Chinese COVID-19 frontline workers (29); and ∆ is precision, with a value of 0.8.

### 2.2. Measures

The questionnaire consisted of six components; (a) socio-demographic data, (b) the short form of the Posttraumatic Growth Inventory (PTGI-SF) assessing PTG, (c) the Kessler Psychological Distress Scale (K6) to assess psychological distress, (d) the General Anxiety Disorder-7 (GAD-7) to assess anxiety, (e) the Patient Health Questionnaire (PHQ-9) to identify depressive symptoms, and (f) the Explanatory Model Interview Catalog Stigma Scale (EMIC-SS) to assess perceived stigma experience. The participants were required to complete the questionnaires 6 months post-hospitalization.

### 2.3. Outcome variables

Posttraumatic growth: PTGI is an instrument used to assess the level of PTG or a positive change in a person that occurs following traumatic events. The scale consists of five factors: personal strength, spiritual change, new possibilities in life, appreciation of life, and relating to others (14). PTGI-SF is a shorter version of the original PTGI, which operates with less information (30) and consists of 10 items. The higher the PTGI-SF score, the higher the level of PTG in the individual being assessed.

A Malay version of the PTGI-SF was translated and validated in a Malaysian population. It demonstrated good internal consistency, with a Cronbach’s alpha of 0.887 (31). In this study, Cronbach’s alpha of the PTGI-SF Malay was 0.966. However, PTGI-SF does not have cut-off values to classify the perceived stigma level as low, moderate, or high, nor lower and upper limits.

### 2.4. Explanatory variables

- Psychological Distress: K6 is a six-item self-rated psychological screening instrument developed by Kessler to assess psychological distress (32). A cut-off point of ≥5 reflects moderate psychological distress, which may warrant mental health treatment (33). The Malay version of the K6 was validated in a Malaysian population, and it exhibited a Cronbach’s alpha of 0.859 (34). In this study, the re-determined Cronbach’s alpha of the Malay version was 0.696.
- Anxiety: GAD-7 is a seven-item questionnaire used to assess generalized anxiety symptoms (35). It is widely administered in research and clinical settings with a recommended cut-off point of ≥10 as an indication of an anxiety disorder (36). This questionnaire has also been validated in the Malay language and reported a Cronbach’s alpha of 0.74 (37). The Cronbach’s alpha of the Malay version in this study was 0.918.
- Depression: PHQ-9 is a nine-item self-administered questionnaire used for screening depressive symptoms. The recommended cut-off point of ≥10 indicated the presence of major depression (38). A validated and reliable Malay version of PHQ-9 is available with a reported Cronbach’s alpha of 0.67 (39). In this study, Cronbach’s alpha of the Malay version was 0.815.
- Stigma experience: The Explanatory Model Interview Catalog (EMIC) stigma scale has been extensively used to measure the degree of perceived stigma against infectious diseases worldwide. The cross-cultural adaptation of EMIC has been documented for diseases such as leprosy, tuberculosis, onchocercal skin disease, leishmaniasis, HIV/AIDS, and COVID-19. Recently, EMIC underwent adaptation and validation among Malaysian COVID-19 patients with an acceptable internal consistency (Cronbach’s alpha of 0.727) (40). Hence, the EMIC stigma scale was used to measure the perceived stigma of COVID-19 in this study.

The EMIC stigma scale is a 15-item self-rated scale originally designed to specifically measure the degree of stigma among patients with leprosy. As COVID-19 is also a disease that requires isolation, the tool is applicable to this study, whereby the higher the score, the higher the level of perceived stigma. Previous studies have demonstrated good internal consistency with a Cronbach’s alpha of 0.897 and 0.88 in EMIC stigma scale items studied in Hong Kong (41) and Ghana (42), respectively. However, EMIC does not have cut-off values to classify the perceived stigma as low, moderate, or high levels and does not have lower or upper limit cut-off values.

- Socio-demographic and personal characteristics data considered for the study include age, sex, occupation, household income, ethnicity, marital status, education status, presence of medical illness, and the presence of counseling-seeking behavior. The participants’ age was recorded as a continuous variable. The answer options for sex were male and female, while the response options for occupation were employed, retired and unemployed, or student. Response options for household monthly income were categorized based on the Malaysian socioeconomic classification of <RM 5,000 (equal to <USD 1,117 based on the currency conversion rate at the time of writing), RM 5,000–RM 10,000, and >RM 10,000, which are also reflective of the bottom 40% (B40), middle 40% (M40), and top 20% (T20) household earners, respectively (43). Ethnicity response options were Malay and non-Malay and marital status options were married, single, divorced, or separated. The options for education status were primary, secondary, or tertiary education. Response options for medical illness presence and counseling-seeking behavior were either yes or no.

### 2.5. Statistical analysis

Statistical analyses were performed using the Statistical Package for Social Sciences, Version 26 (SPSS 26; SPSS Inc., Chicago, Illinois). Descriptive statistics were reported for demographic, personal, and clinical factors, as well as scores for EMIC, K6, PHQ-9, GAD-7, and PTGI-SF. The categorical variables were presented as frequencies and percentages. Continuous variables were presented as median and interquartile range, as the variables were non-normally distributed. There were no missing data. In order to achieve the main objective of the study, a multivariable general linear model with bootstrapping with 2000 replications was computed to assess the association between demographic; clinical; and EMIC, K6, PHQ-9, GAD-7 (independent variables) and PTGI-SF (dependent variable). The statistical significance was set at *p* < 0.05 for the multivariable general linear model.

### 2.6. Ethics statement

This study received approval from the Medical Research Committee of Universiti Kebangsaan Malaysia Medical Cenetr (UKM PPI/111/8/JEP-2020-352) and the Medical Research and Ethics Committee of the Ministry of Health Malaysia (NMRR-20-1,288-55,105). The study abides by the regulations of the 1964 Declaration of Helsinki and its subsequent amendments.

## 3. Results

### 3.1. Respondent characteristics

A total of 152 out of 219 COVID-19 survivors invited to participate completed the online survey, with a response rate of 69.4%. The high drop-out rate was expected as the patients were approached after they had been discharged from hospital. The sociodemographic details, clinical characteristics, and scores from the EMIC, K6, PHQ-9, GAD-7, and PTGI-SF tools are summarized in Table 1.

**Table 1:** Sociodemographic and clinical characteristics of participants.

Variables	N	%
Age	34.0^#^	19.0^$^
Sex:		
Female	54	35.5
Male	98	64.5
Ethnicity:		
Malay	125	82.2
Non-Malay	27	17.8
Employment status:		
Retired	10	6.6
Unemployed/housewife/		
students	27	17.8
Employed	115	75.6
Monthly household		
income:		
B40 (<RM 5,000)	95	62.5
M40 (RM 5000—RM 10,000)	43	28.3
T20 (>RM 10,000)	14	9.2
Marital status:		
Married	83	54.6
Single/divorced/separated	69	45.4
Education status:		
Primary education	8	5.3
Secondary education	50	32.9
Tertiary education	94	61.8
History of psychiatric illness:		
No	146	96.1
Yes	6	3.9
History of medical illness:		
No	113	74.3
Yes	39	25.7
Counseling-seeking behavior:		
No	86	56.6
Yes	66	43.4
Median total EMIC score	5.5^#^	8.0^$^
Median total K6 score	10.0^#^	50.0^$^
Median total PTGI-SF score	40.0^#^	16.0^$^
Depression status of participants		
No	139	91.4
Yes	13	8.6
Median total PHQ-9 score	2.0^#^	4.0^$^
Anxiety status of participants		
No	147	96.7
Yes	5	3.3
Median total GAD-7 score	0.5^#^	5.0^$^

# = median, $ = interquartile range.

### 3.2. Associations between socio-demographic and clinical characteristics, perceived stigma, and psychological sequelae in COVID-19 patients 6 months after discharge

Table 2 shows a multivariable general linear model (with bootstrapping of 2000 replications) between various factors and total PTG-SF scores. The factors which significantly predicted PTG include Malay ethnicity (*B* = 12.767, 95% CI = 7.541 to 17.993, *p* < 0.001), higher perceived stigma scores (*B* = 0.367, 95% CI =. 0.041 to 0.691, *p* = 0.026), retirees (*B* = −12.060, 95% CI = −21.310 to −2.811, *p* = 0.011), and those with a history of medical illness (*B* = 4.971, 95% CI = 0.096 to 9.845, *p* = 0.046). Malay participants reported a PTG score 12.767 points higher than non-Malay participants. On the contrary, retired COVID-19 patients registered PTGI-SF scores that were 12.060 points lower than employed patients. Participants with a history of medical illness reported a PTG score that was 4.971 points higher compared to those without. Finally, an increase in the perceived stigma score by 1 unit was associated with an increase of 0.367 units in the PTGI-SF score, when all the other independent variables (demographics, clinical variables, PHQ-9, GAD-7, K6 scores) were held constant.

**Table 2:** The association between individual socio-demographic and clinical characteristics, EMIC, PHQ-9, GAD-7, K6 scores, and total PTGI-SF among COVID-19 patients 6 months post-hospitalization.

Variables	*B*	BCa 95% confidence interval		Standard error	Value of *p*
		Lower	Upper		
Age	0.002	−0.234	0.239	0.121	0.985
Sex:					
Male	Reference	1.224	7.121	1.224	0.166
Female	2.949				
Ethnicity:					
Non-Malay	Reference	7.541	17.993	2.667	<0.001*
Malay	12.767				
Employment status:					
Employed	Reference	−21.310	−2.811	4.719	0.011*
Retired	−12.060	−4.738	5.748	2.675	0.850
Unemployed/housewife/	0.505				
students					
Monthly household					
income:					
>RM 10,000	Reference	−4.602	10.700	3.904	0.435
<RM 5,000	3.049	−6.445	8.553	3.826	0.783
RM 5,000—RM 10,000	1.054				
Marital status:					
Married	Reference	−6.784	3.076	2.515	0.461
Single/divorced/separated	−1.854				
Education status:					
Tertiary education	Reference	−14.426	3.700	4.624	0.246
Primary education	−5.363	−4.911	4.119	2.304	0.863
Secondary education	−0.396				
History of psychiatric					
illness:					
No	Reference	−8.661	11.068	5.033	0.811
Yes	1.203				
History of medical illness:					
No	Reference	0.096	9.845	2.487	0.046*
Yes	4.971				
Counseling-seeking behavior:					
No	Reference	−0.469	7.572	2.051	0.083
Yes	3.552				
Total PHQ-9 score	−0.536	−1.391	0.320	0.436	0.220
Total GAD-7 score	−0.347	−1.441	0.746	0.558	0.533
Total K-6 score	0.440	−0.326	1.206	0.391	0.260
Total EMIC score	0.367	0.043	0.691	0.165	0.026*

* Statistical significance at *p* < 0.05.

## 4. Discussion

This study investigates the level of PTG and its associations with stigma, psychological complications, and sociodemographic factors among COVID-19 patients 6 months after discharge. The study was conducted after the Malaysian government’s decision to lift the Movement Control Order. The study findings confirm the hypothesis that a higher perceived stigma score significantly predicts PTG. In addition, those of Malay ethnicity with a history of medical illness were also significantly associated with higher PTG, but retirees were associated with lesser PTG. No significant associations were found between psychological complications (i.e., anxiety, depression, psychological distress) and PTG.

Relative to other populations measured using the same instrument (the PTGI-SF), the degree of reported PTG in this study was comparable. For example, the median PTG reported in this study was 40.0, while the median PTG reported in a study of Malaysian cancer patients was between 30.0 and 37.5 (44). Moreover, another two studies on PTG in Malaysian subjects also reported similar degrees of PTG, with the mean ranging from 39.3 to 39.87 (16, 40). While the nature of the trauma in this study was infection as opposed to malignancy, as in the other studies, it is notable that the degrees of PTG across these studies were comparable, further supporting the role of COVID-19 as a traumatic stressor.

This study demonstrates that perceived stigma is associated with higher PTG. Another study in China on COVID-19 survivors 6 months post-discharge also revealed self-stigma as one of the factors positively associated with PTG, in addition to social support and mental health care access during hospitalization (45). As COVID-19 survivors are present globally, stigma has become an additional issue to deal with among communities. Due to stigma, COVID-19 survivors reported being stalked, avoided, and even abandoned by family members (46). Being stigmatized is also a negative experience that may lead to psychological trauma, but it is also probable that affected individuals would eventually engage in a positive adaptation process via cognitive re-appraisal of a traumatic experience. This proactive response would then enhance the development of PTG (15).

An important finding indicates that although negative psychosocial sequelae, such as perceived stigma, contribute to higher PTG, it does not imply that the perceived stigma among COVID-19 survivors should be left unmanaged. This is given the evidenced curvilinear across-time relationship between PTSD and PTG, whereby the increasing degree of trauma initially contributes to increasing PTG, but after a certain level, this effect changes. A further increase in trauma beyond the threshold causes an overwhelming degree of psychological sequelae to occur and may depreciate PTG as they may interfere with the search for meaning outside the traumatic experience (47, 48). A similar occurrence has been demonstrated in a study involving survivors of an earthquake in China, whereby survivors who had experienced moderate levels of disaster exposure had the highest PTG scores, but having higher or lower exposure led to a reduction in PTG scores (49).

As an overwhelming degree of trauma may hinder positive psychological sequelae, close monitoring of the level of stigma experienced is important. They need to be monitored closely to ensure they fall within the mild and moderate levels that may promote PTG. Highly intense trauma leads to failure in the cognitive reprocessing of an event and disrupts the search for new perspectives and the narrative development required in the process of developing PTG (50). Moreover, a recent longitudinal study demonstrated that PTG was associated with COVID-19 patients receiving psychological consultation after discharge (51).

This study also found that Malays had a higher PTG level. As all Malays in Malaysia are Muslims (52), the positive development of PTG among them might be explained by the concept of spiritual coping. Spiritual coping is based on religious beliefs, practices, and teachings (Abu-Raiya and Pargament, 2015 (53). A constructive feeling may be developed when an individual is able to find a sense of spiritual connection. The connection occurs by reflecting on a secure relationship with God to achieve five basic goals, namely meaning, control, comfort, intimacy, and life transformation (54). Empirical studies have demonstrated that spirituality is fundamental in the meaning-making framework, especially following traumatic events that have shattered initial assumptions about oneself and the world, fostering the search for new meaning in life (55). Eventually, a new assumptive world is developed, and this better state helps promote better PTG (56). In another study conducted among Indonesian tsunami survivors, positive spiritual coping among Muslim survivors predicted PTG (57).

As PTG is shown to arise from highly stressful situations (58), retired participants demonstrated lower PTG scores, which suggests that retirees lacked work stress. It was also probable that employed participants were distracted by daily meaningful and challenging activities that kept them occupied. These activities assisted them in living through difficult circumstances. In Malaysia, the retirement age is 60 (59); hence, nearly all the retired participants were from the elderly age group. Additionally, participants of older age were more ‘accepting’ of the stressful condition as a natural occurrence in life compared to the younger respondents; hence, the younger group demonstrated a reduced likelihood of developing PTG (60).

In addition, we discovered that participants with co-morbid medical illnesses had higher PTG scores compared to those without a history of medical illness. A previous study reported that the diagnosis of a life-threatening illness can be perceived as an extremely stressful and traumatic experience, but many survivors also reported experiencing various positive changes, referred to in empirical literature as PTG (61).

The current study did not find a significant association between the level of anxiety, depression, and psychological distress and PTG. It is concluded that PTG is a unique positive psychology, which may co-exist with other psychological conditions such as depression and anxiety. Previous prospective studies on PTG have also drawn similar conclusions (62, 63).

### 4.1. Limitations

A few limitations are outlined in this study. Firstly, the cross-sectional approach does not allow the determination of a causal relationship between the variables explored and PTG over time. Secondly, the respondents were not randomly sampled, which limited the complete representation of COVID-19 patients, thus restricting the generalization of the outcome. Thirdly, although sufficient according to the sample size calculation, the number of participants was relatively small. In addition, the lack of data on the clinical stages of COVID-19 was a confounding factor in this study.

### 4.2. Implications of study findings

This study was the first in Malaysia to explore PTG among COVID-19 patients. The data suggest that spiritual coping is potentially an appropriate psychosocial intervention that facilitated COVID-19 survivors to deal with traumatizing experiences. Spiritual coping led to a better spiritual connection with God, and this helped patients to positively live or move on after trauma. Based on the community data, it is also recommended that retirees be more involved in daily activities, as this may enhance their PTG level and improve their mental well-being.

Based on the data collected and understanding of limitations, a longitudinal version of the study is proposed to better elucidate variables that have a causal impact on PTG. The inclusion of a relationship analysis between the development of PTG and the extent of perceived stigma experienced by COVID-19 survivors would have added value to the study.

## 5. Conclusion

In conclusion, experiencing stigma contributed to the PTG of COVID-19 survivors in addition to other sociocultural factors that are influenced by ethnicity, history of medical illness, religion, and work status. No associations were found between PTG and depression and anxiety. The findings provided valuable insights and understanding of the predictors of PTG that may suggest incorporating various psychologically beneficial interventions to improve the well-being of COVID-19 survivors.

## Data availability statement

The original contributions presented in the study are included in the article/Supplementary material, further inquiries can be directed to the corresponding author.

## Ethics statement

The studies involving human participants were reviewed and approved by Medical Research Committee of Universiti Kebangsaan Malaysia Medical Center (UKM PPI/111/8/JEP-2020-352) and the Medical Research and Ethics Committee of the Ministry of Health Malaysia (NMRR-20-1,288-55,105). The patients/participants provided their written informed consent to participate in this study.

## Author contributions

All persons who meet authorship criteria are listed as authors, and all authors certify that they have participated sufficiently in the work to take public responsibility for the content, including participation in the concept, design, analysis, writing, or revision of the manuscript. Furthermore, each author certifies that this material or similar material has not been and will not be submitted to or published in any other publication before its appearance in the Frontiers. NA: acquisition of data, analysis and/or interpretation of data, drafting the manuscript. NN: conception and design of the study, revising the manuscript critically for important intellectual content. ML: analysis and/or interpretation of data, revising the manuscript critically for important intellectual content. NM: conception and design of the study. NA: acquisition of data. MA: acquisition of data. SD: acquisition of data. All authors contributed to the article and approved the submitted version.

## Conflict of interest

The authors declare that the research was conducted in the absence of any commercial or financial relationships that could be construed as a potential conflict of interest.

## Publisher’s note

All claims expressed in this article are solely those of the authors and do not necessarily represent those of their affiliated organizations, or those of the publisher, the editors and the reviewers. Any product that may be evaluated in this article, or claim that may be made by its manufacturer, is not guaranteed or endorsed by the publisher.
